# Latitudinal Gradient in Otolith Shape among Local Populations of Atlantic Herring (*Clupea harengus* L.) in Norway

**DOI:** 10.1371/journal.pone.0130847

**Published:** 2015-06-23

**Authors:** Lísa Anne Libungan, Aril Slotte, Åse Husebø, Jane A. Godiksen, Snæbjörn Pálsson

**Affiliations:** 1 Department of Life and Environmental Sciences, University of Iceland, Reykjavík, Iceland; 2 Institute of Marine Research, Bergen, Norway; 3 Hjort Centre for Marine Ecosystem Dynamics, Bergen, Norway; Aristotle University of Thessaloniki, GREECE

## Abstract

Otolith shape analysis of Atlantic herring (*Clupea harengus*) in Norwegian waters shows significant differentiation among fjords and a latitudinal gradient along the coast where neighbouring populations are more similar to each other than to those sampled at larger distances. The otolith shape was obtained using quantitative shape analysis, the outlines were transformed with Wavelet and analysed with multivariate methods. The observed morphological differences are likely to reflect environmental differences but indicate low dispersal among the local herring populations. Otolith shape variation suggests also limited exchange between the local populations and their oceanic counterparts, which could be due to differences in spawning behaviour. Herring from the most northerly location (69°N) in Balsfjord, which is genetically more similar to Pacific herring (*Clupea pallasii*), differed in otolith shape from all the other populations. Our results suggest that the semi-enclosed systems, where the local populations live and breed, are efficient barriers for dispersal. Otolith shape can thus serve as a marker to identify the origin of herring along the coast of Norway.

## Introduction

Atlantic herring (*Clupea harengus*, Linneaus 1758), being one of the economically most important fish species, has been a subject of several studies on population structure [1–8]. A relatively low level of genetic differentiation has been found among isolated local populations which may overlap geographically during feeding migrations [2–6,9–12]. Genetic markers have shown uniformity among herring occupying the offshore waters of the Northeast Atlantic [13,14] and over large geographical distances [1,15,16]. However, recent studies on population genomics have revealed clear differentiation among Baltic Sea herring [5] and genetic differences have also been found between the geographically isolated local herring populations in Norway, the Lake Landvik herring and herring from Trondheimsfjord, Lindåspollene and Lusterfjord [1] and also within Balsfjord and Trondheimsfjord [17,18]. Studies on Atlantic herring have further revealed the plasticity and high level of adaptability of the species [19] as observed in heterogeneity in life history, morphology and behaviour [20], and reported population differences which are evident in otolith shape descriptors but have not been detected with genetic markers [8].

An indented coastline, such as found in Norway, provides an excellent model system for evaluating the effects of geographic barriers on patterns of isolation in marine fish populations. The fjord system presents different hydrographic conditions than found in the open ocean. Within fjords, the conditions can be uniform and stable due to stratification of the water masses where the upper layers have comparatively low salinity owing to freshwater carried into the sea by rivers [21]. Thermal stratification in the water column is for example known to influence maturation and time of spawning for local Atlantic herring populations in Norway [22].

Several local herring populations in Norway have been identified based on biological characteristics and geographical distribution, such as the Balsfjord, Lysefjord and Østerbø herring [23], Borge poll herring [24], Lindåspollene herring [25], Lusterfjord herring [21], Lake Landvik herring [26], Lake Rossfjord herring [27] and Trondheimsfjord herring [28,29]. The local herring populations are thought to complete their entire life-cycle within fjords [21], lakes [26] and semi-enclosed coastal systems [22] and differ from their oceanic counterparts by having small population sizes, a shorter life cycle, low vertebral number, slower growth rate [21], and smaller size-at-age [30,31], but also in having higher relative fecundity since local populations do not migrate over long distances and therefore invest less energy into growth and more into egg production than oceanic populations [27,32–34]. As the herring larvae have limited swimming capabilities, where they can only travel short distances of 14.7–16.1 mm s^-1^ as measured for larvae at the age of 34 days post-hatch [35], and they are not carried passively with the coastal current as most fry of the oceanic populations [36–39], it is likely that they retain close to their site of spawning in semi-enclosed ecosystems. In addition to the local herring populations in Norway, there are two oceanic herring populations; the Norwegian spring-spawners which is highly migratory and disperses all over the Norwegian Sea, and the Norwegian autumn-spawners which is thought to be mainly around Lofoten [40] and is managed as part of the Norwegian spring-spawners. Where the Norwegian spring-spawners overlap geographically with local herring, the first year cohort is known to utilize fjords as an overwintering area and then migrate out of the fjord during the summer to feed [41–43]. The extent of interaction and reproduction between the Norwegian spring-spawners and the local populations is not fully explored. However, the interaction between the Norwegian spring-spawners and Lindåspollene herring was studied over a 50 year period and results showed the latter population to change in several life-history traits including length-at-age, length at first maturity and longevity when the Norwegian spring-spawners were spawning at the same time and in the same semi-enclosed coastal region [7], confirming that the Norwegian spring-spawners do interbreed at least with some of the local populations.

Otolith shape analysis has been widely used with success in stock identification of various marine fish species with high gene flow, such as cod [44], haddock [45], anchovy [46], horse mackerel [47,48] and herring [8,49]. Otolith shape is markedly population specific, but also shows intra-specific geographic variation in relation to environmental factors [8,26,50,51]. Since morphometric characters are modified by the environment, they can indicate reproductive isolation if the characters are different between spawning aggregations [52].

The aim of this study was to investigate the structure of local herring populations along the Norwegian coastline using otolith shape, which is a known population marker for Atlantic herring [8], to describe how discrete and diverse these smaller populations are and if so whether there were any signs of dispersal among neighbouring and more distant local populations. The northernmost population, which was sampled in Balsfjord, is known to be similar to Pacific herring (*Clupea pallasii*, Valenciennes 1847) in vertebrae number, spawning behaviour [17] and genetics [53]. A second aim of the study was to compare otolith shape between local populations and neighbouring oceanic populations.

## Material and methods

### Sampling

**Fig 1:**
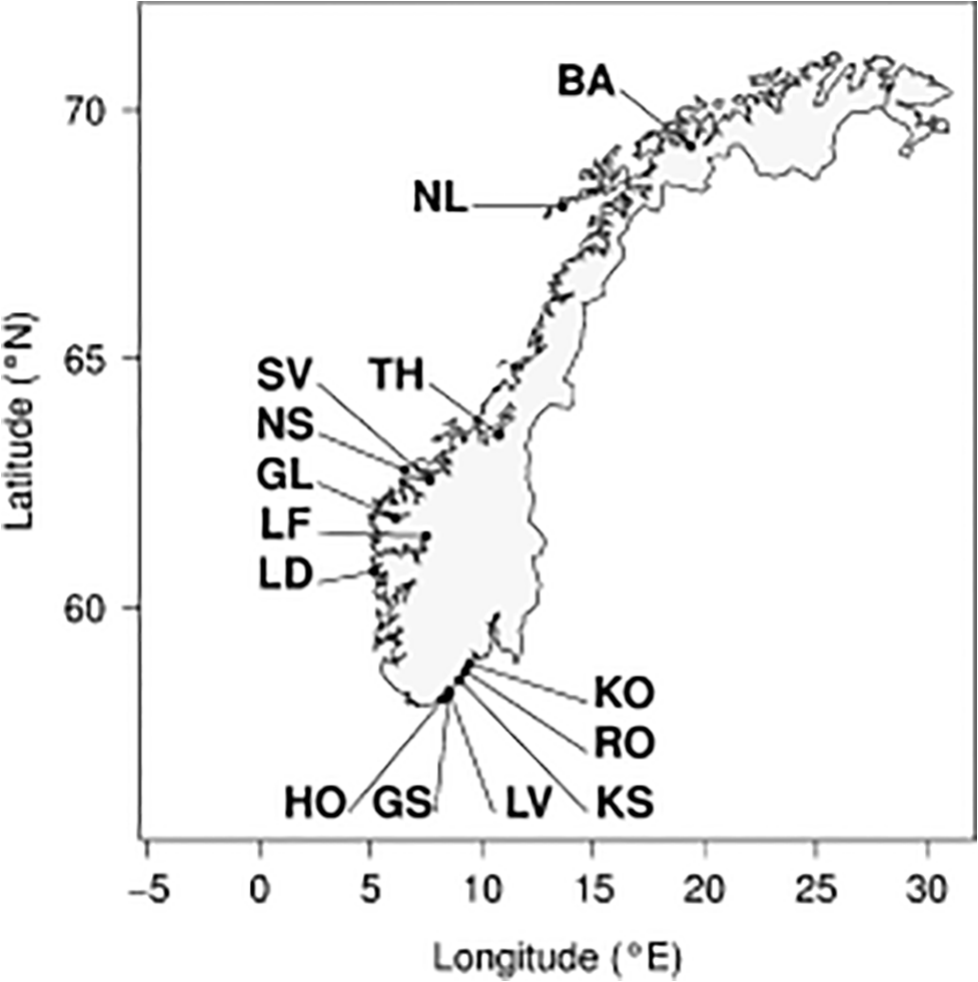
Herring sampling areas along the coast of Norway. Local populations from southern Norway are KO: Kragerø, RO: Risør, KS: Kilsund, LV: Lake Landvik, GS: Grimstad, HO: Hovåg. From western Norway LD: Lindåspollene, LF: Lusterfjord, GL: Gloppen, SV: Sykkulven, TH: Trondheim. From northern Norway BA: Balsfjord. The two oceanic populations, NS: Norwegian spring-spawners and NL: Norwegian-autumn spawners are also shown (see Table 1 for further details). Latitude (°N) is shown on the y-axis, and longitude (°E) on the x-axis.

Herring were sampled during the period of 2005–2014 from 14 different spawning grounds with purse-seiners from fjords, semi-enclosed coastal regions, Lake Landvik and the open ocean (oceanic populations) clockwise from southern (Kragerø, 58.88N, 9.43E) to northern Norway (Balsfjord, 69.27N, 19.35E, Fig 1, Table 1). The local populations from southern Norway were sampled at Kragerø, Risør, Kilsund, Lake Landvik (a brackish lake connected to the ocean), Grimstad and Høvåg. From western Norway, samples were obtained from Lindåspollene, Lusterfjord (200 km from the coastline), Gloppen (80 km from the coastline), Sykkulven and Trondheim. The oceanic populations were the Norwegian spring-spawners, sampled at their main spawning grounds at Møre and the Norwegian autumn-spawners from Lofoten [40]. Sampling areas and time of sampling were selected based on knowledge of spawning behaviour of herring at each location, ensuring individuals sampled belonged to the spawning stock of that site.

**Table 1:** Samples of Atlantic herring from fourteen locations (Area) shown clockwise from Kragerø in southern Norway to Balsfjord in northern Norway along the coast of Norway (see also Fig 1).

									Length			n			
Area	ID	Lat	Lon	N/W/S	System	Month	Year	Spawn	3–5y	6–8y	9–12y	3–5y	6–8y	9–12y	n_tot_
**Kragerø**	KO	58.88	9.43	S	Fjord	Mar	2006	+	28.1 [24.5–32.0]	30.1 [27.0–32.5]		42	38		80
**Risør**	RO	58.73	9.24	S	Fjord	Nov	2005, 2006	+	28.4 [23.0–31.5]			60			60
**Kilsund**	KS	58.55	8.98	S	Fjord	Jan	2012	+	27.8 [26.0–30.0]			32			32
**Lake Landvik**	LV	58.33	8.50	S	Lake	June	2012	+	26.9 [24.0–30.5]	28.9 [28.0–30.0]	29.8 [28.0–31.5]	132	8	20	160
**Grimstad**	GS	58.28	8.52	S	Fjord	Feb-May	2012	+	28.1 [23–32.5]	31.1 [25.0–34.0]	31.8 [29.0–34.0]	290	66	27	383
**Høvåg**	HO	58.17	8.25	S	Fjord	Feb	2012	+	28.9 [27–31.5]	31.6 [29.5–37.0]	32.7 [31.5–34.5]	15	19	14	48
**Lindås**	LD	60.73	5.15	W	Fjord	Mar	2010	+	30.0 [28.0–32.5]	32.4 [31.0–34.5]	32.6 [31.0–36.0]	3	10	27	40
**Lusterfjord**	LF	61.44	7.48	W	Fjord	Nov	2011	-	18.4 [16.0–22.5]	19.5 [19.5–19.5]		89	1		90
**Gloppen**	GL	61.80	6.12	W	Fjord	Feb	2009, 2010, 2012, 2013	+	22.0 [19.5–24.5]	22.6 [20.5–26.5]	23.8 [21.0–26.0]	34	50	9	93
**Møre**	NS	62.52	5.23	W	Ocean	Feb	2010	+	30.6 [29.0–32.5]	32.6 [29.0–34.5]		8	78		86
**Sykkulven**	SV	62.56	7.64	W	Fjord	Nov	2012, 2013	-	27.7 [25.0–33.0]	28.7 [27.5–30.0]	28.0 [28–28]	42	19	1	62
**Trondheim**	TH	63.47	10.75	W	Fjord	Mar	2010	-	27.1 [23.0–30.0]	26.7 [25.0–28.0]	27.5 [26–30]	8	19	64	91
**Lofoten**	NL	68.06	13.60	N	Ocean	Aug	2010	+	30.6 [27.0–34.5]	33.6 [31.5–36.0]		17	16		33
**Balsfjord**	BA	69.27	19.35	N	Fjord	Apr	2012, 2014	+	21.8 [17.5–26.5]	26.0 [24.5–27.5]		57	26		83

ID: Population abbreviation, Lat: latitude (N), Lon: longitude (E), N/W/S: N: populations in northern Norway, W: populations in western Norway, S: populations in southern Norway, System: type of habitat where the herring were sampled, Month: month of spawning, Year: sampling year, Spawn: some in spawning condition (+), none in spawning condition (-), Length: mean length in cm and length range for each age range 3–5 years, 6–8 years, 9–12 years, n: total number of samples for each age range, n_tot_: total number of samples from each area.

To test for temporal effects in otolith shape, herring in Balsfjord, Gloppen, Risør and Sykkulven were sampled for 2–4 years (Table 1). Total length (cm) was recorded for each fish and maturity stage according to an 8-point scale: immature = 1 and 2, maturing = 3 to 5, running/spawning = 6, spent = 7, recovering/resting = 8 [54]. The sagittal otoliths were removed, washed in clean water and stored in plastic trays. All fish were aged from their scales using standard ageing techniques [55].

The Institute of Marine Research (IMR), which is responsible for monitoring herring and giving advice to fisheries managers in Norway, have permission to sample herring at any location along the Norwegian coast by the Directorate of Fisheries, Bergen, Norway. In addition, any person in Norway has by law permission to conduct recreational fisheries on herring at these sites using gill nets. The samples used in this study stem from both trawl hauls using IMR's research vessel, IMR's gillnet sampling as well as samples collected by recreational fishermen, all sampled within Norwegian regulations and laws. There is, however, one exception from this general permission to sample herring, and that is the Lake Landvik location. Given that this is an inland lake connected to the sea through an artificial channel, other rules apply. Here, a special permission to sample herring with gillnets inside Lake Landvik and the connected fjord system was granted by the County Governor of Aust-Agder, Arendal, Norway. Our study did not involve any endangered or protected species.

### Image and data analysis

A digital image of each otolith was captured using either a Leica M60 stereomicroscope with a Leica DFC450 camera and the software Leica Application Suite (LAS Version 4.5) (Leica Micro-systems, Wetzlar, Germany, www.leica-microsystems.com) or a Leica MZ95 stereomicroscope (Leica Micro-systems) with an Evolution LC-PL A662 camera (MediaCybernetics, Maryland, USA) using the software PixeLINK 3.2 (www.pixelink.com). All statistical analysis were conducted with R [56] using the R packages ade4 [57], shapeR [58] and vegan [59].

### Visualizing the main shape features

The variation in otolith shape was examined by plotting the mean shape of each population using the shapeR package [58]. To inspect how the variation in the Wavelet coefficients which represent the otolith shape is dependent on the position along the outline, the mean and standard deviation of the coefficients was plotted against the angle using the gplots package [60]. To quantify the differences among populations, the proportion of variation among groups (the intraclass correlation, ICC), was calculated along the outline of the otolith.

### Multivariate analysis of shape

Following a method implemented in [8], otolith images were read into R and outlines collected from the digital images using the shapeR package [58]. The shape of each otolith was recorded as a matrix of x and y coordinates. Equally spaced radii were drawn from the otolith centroid to the otolith outline and independent Wavelet shape coefficients, which represent the otolith shape, were obtained by conducting a discrete Wavelet transform to the equally spaced radii using the wavethresh package [61].

Temporal stability in otolith shape was analysed within sampling areas for the regions with more than one sampling year to see if it was possible to combine the samples (Tables 1 and 2) by applying Canonical Analysis of Principal coordinates (CAP) [62] and an ANOVA like permutation test to assess the significance of constraints using 2000 permutations with the vegan package in R [59]. Otolith shape was then compared among populations with overall tests and also by applying a priori comparisons to test for regional differences, also using the CAP and the ANOVA like permutation test and to evaluate differences between age classes and the interaction of age and geographic origin (Table 3). Age is known to have confounding effects on otolith shape [63], and as interaction between age and geographic origin was significant, the dataset was divided into three age groups: 3–5 years, 6–8 years, 9–12 years. An analysis of covariance (ANCOVA) was performed on the Wavelet coefficients to determine if there was an interaction between the total length of the fish and population. When there was a significant interaction, those coefficients were excluded from the analysis, which resulted in a total of 55 Wavelet coefficients being used in all analysis [45,64,65]. The CAP values for each population at each age were adjusted by taking age as a covariate in the model. Variation for each age group (3–5, 6–8, 9–12 years) at each location was summarised by calculating the variance (Table 4) within populations for each age group, based on pairwise distances between individuals. High variation could result from admixture of populations or developmental variation. Ordination of the population averages along the first two canonical axes (CAP1 and CAP2) were examined graphically with the shape descriptors.

**Table 2:** Temporal stability in otolith shape among populations with more than one sampling year.

Area	Df	Var	*F*	*P*
**Balsfjord**	1	1.20	1.54	0.149
**Gloppen**	3	5.28	1.66	0.051
**Risør**	1	1.733	1.67	0.114
**Sykkulven**	1	1.99	1.54	0.152

Results from ANOVA like permutation tests based on 2000 permutations, df: degrees of freedom, Var: variance, *F*: F-value, *P*: p-value, p<0.05 indicates a significant effect. See Table 1 for further details on the populations.

**Table 3:** Otolith shape compared among all herring populations in the present study.

	3–5 years				6–8 years				9–12 years			
	**df**	**Var**	***F***	***P***	**df**	**Var**	***F***	***P***	**df**	**Var**	***F***	***P***
**All populations**	11	68.25	8.47	0.001	10	0.16	6.43	0.001	5	0.21	5.40	0.001
** BA vs fjord popul.**	1	16.66	21.53	0.001	1	0.05	13.43	0.001				
** West vs South**	1	15.00	19.49	0.001	1	0.03	7.98	0.001	1	0.06	7.40	0.001
** Within West**	2	8.13	5.90	0.001	2	7.55	5.39	0.001	1	4.82	5.30	0.001
** Within South**	5	15.71	4.16	0.001	3	0.05	2.26	0.008	2	0.07	1.36	0.16
** NL vs fjord popul.**	1	7.15	8.95	0.001	1	0.01	4.05	0.003				
** NS vs fjord popul.**	1	5.42	6.77	0.001	1	0.05	16.50	0.001				
**Residual**	860	590.66			338	0.86			155	1.18		

Results from ANOVA like permutation tests based on 2000 permutations, df: degrees of freedom, Var: variance, F: F-value, P: p-value, p<0.05 indicates a significant effect. Results for the three age groups 3–5 years, 6–8 years and 9–12 years are shown separately. Local populations from western Norway are: Sykkulven, Gloppen, Lusterfjord and Lindåspollene and populations from southern Norway are Grimstad, Høvåg, Kragerø, Kilsund, Lake Landvik and Risør. The northern local population was sampled in Balsfjord (BA). The oceanic populations are the Norwegian spring- (NS) and autumn-spawners (NL) (see Table 1 for population ID codes). P<0.05 indicates a significant effect. Empty cells indicate data did not exist for these comparisons.

**Table 4:** Variance within each population for the three age groups 3–5 years, 6–8 years and 9–12 years shown along the Norwegian coast from south (Kragerø) to north (Balsfjord).

Area	ID	3–5y	6–8y	9–12y
**Kragerø**	KO	17.54	5.20	
**Risør**	RO	19.09		
**Kilsund**	KS	39.87		
**Lake Landvik**	LV	17.63	0.67	19.67
**Grimstad**	GS	20.35	10.07	16.23
**Høvåg**	HO	18.72	9.79	7.37
**Lindåspollene**	LD		0.84	19.37
**Lusterfjord**	LF	12.26		
**Gloppen**	GL	13.69	5.25	0.67
**Møre**	NS	14.44	6.45	
**Sykkulven**	SV	31.51	1.45	
**Trondheim**	TH		0.25	16.45
**Lofoten**	NL	32.71	5.99	
**Balsfjord**	BA	18.04	3.70	

Empty cells refer to missing observations.

### The association of shape and geographical distance

To examine the association of otolith shape with respect to geographic distances between sampling areas, matrices with shape distances and geographical distances where calculated. Morphological distances were constructed based on average Euclidean distances based on otolith shape (CAP1 and CAP2) for each population, while the geographical distances between sampling areas were calculated by measuring the distance in km between areas along the coastline from Kragerø in southern Norway to Balsfjord in northern Norway. The association of the distance matrices were evaluated with Mantel tests [66] using the ade4 package in R [57] with 10.000 permutations.

## Results

### Main shape features

**Fig 2:**
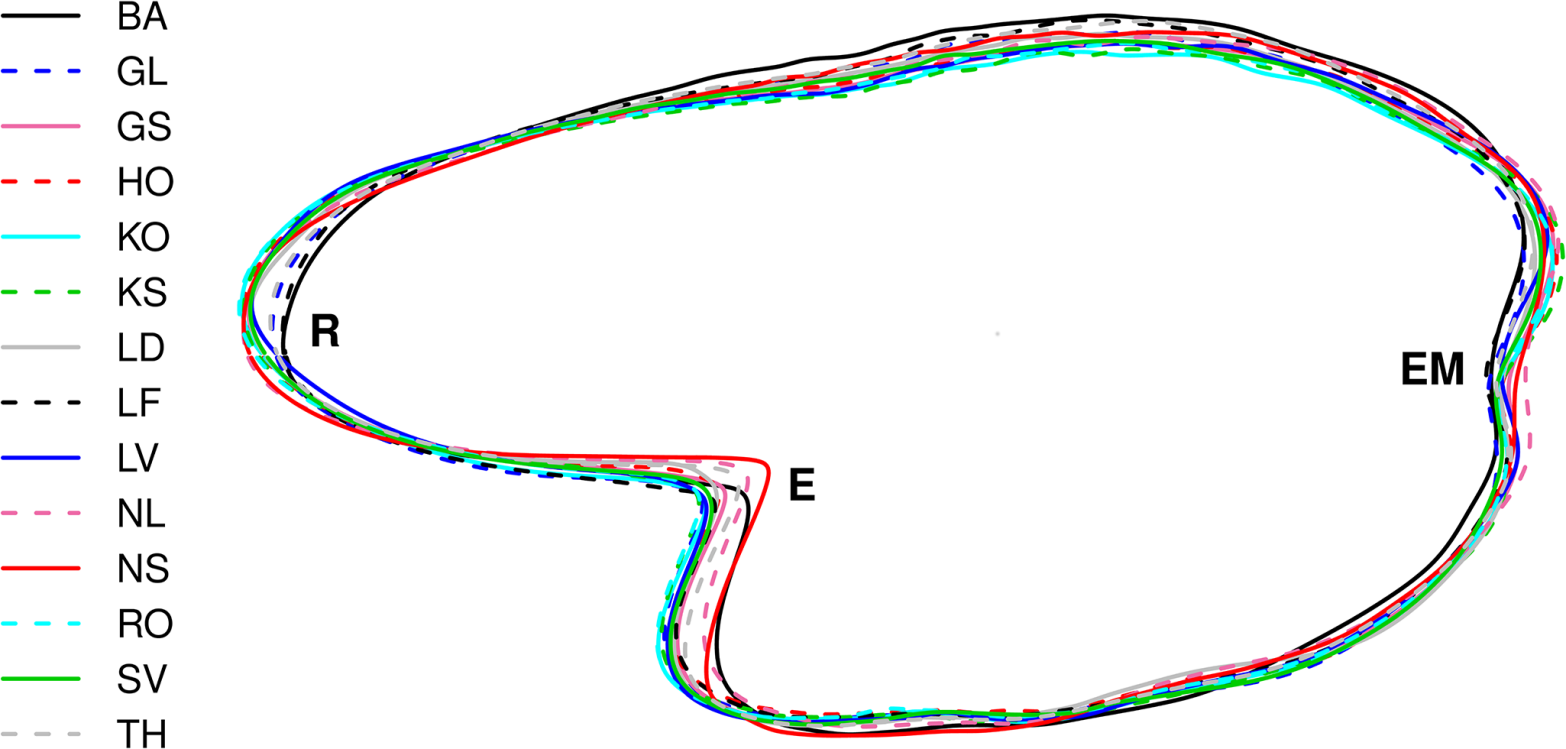
Average shape of all otoliths for fourteen herring populations in Norway. The areas are: BA: Balsfjord, GL: Gloppen, GS: Grimstad, HO: Hovåg, KO: Kragerø, KS: Kilsund, LD: Lindåspollene, LF: Lusterfjord, LV: Lake Landvik, NL: Lofoten, NS: Møre, RO: Risør, SV: Sykkulven and TH: Trondheim in Norway for three age groups (see Table 1 for further details). The excisura major (E), rostrum (R) and excisura minor (EM) are marked.

**Fig 3:**
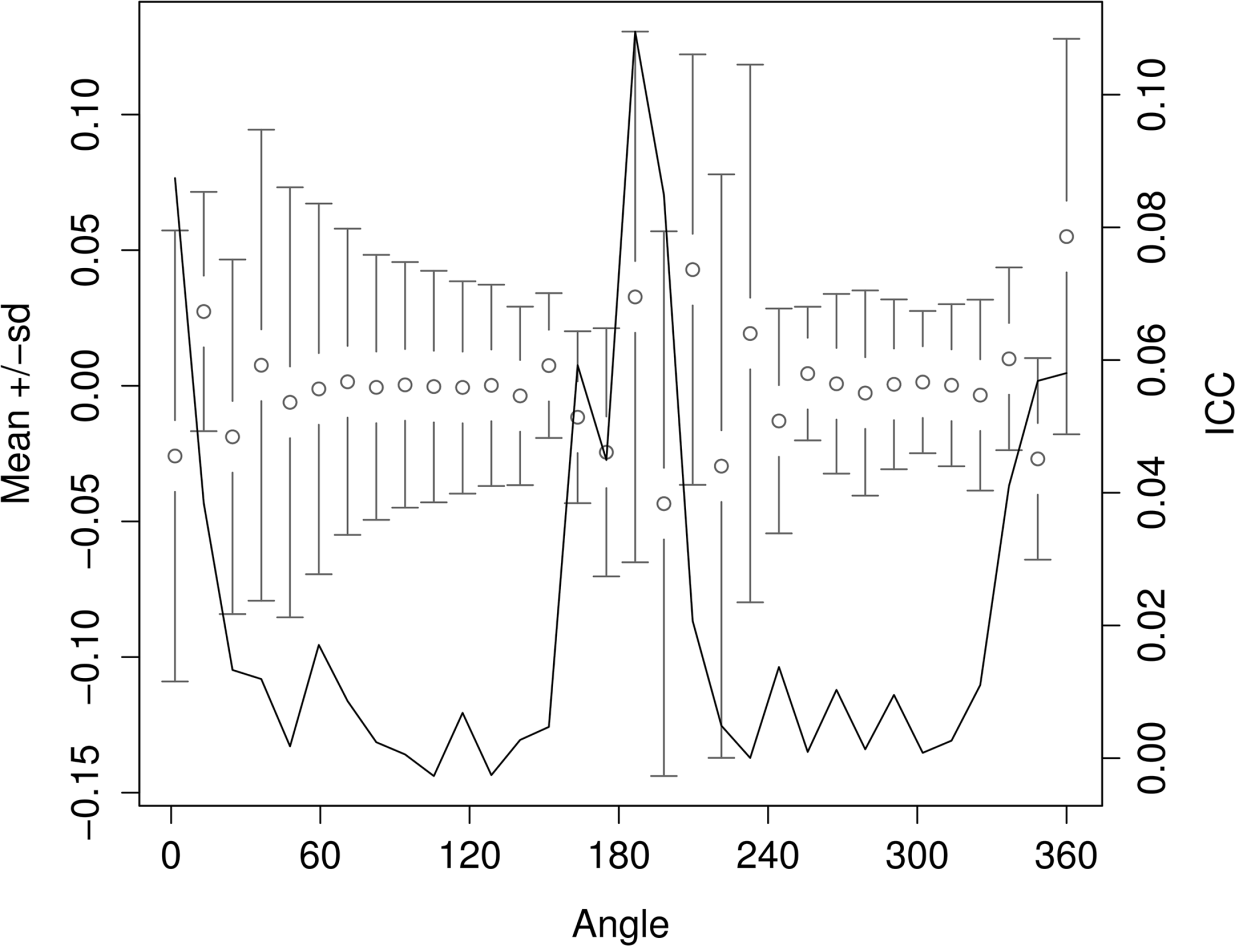
Mean and standard deviation (sd) of the Wavelet coefficients (grey) representing shape for all combined otoliths and the proportion of variance among herring populations or the intraclass correlation (ICC, black solid line). The horizontal axis shows angle in degrees (°) based on polar coordinates where the centroid of the otolith is the center point of the polar coordinates.

Otolith shape differed among all of the populations in the study, as reflected in the differences in the mean shape (Fig 2) and high level of between population variation in the Wavelet coefficients (ICC) for these regions on the otolith outline at 0–20° and 170–190° (Fig 3).

### Multivariate analysis of otolith shape

Samples obtained at two or more years from the same area did not differ in otolith shape (p>0.05, Table 2) and were therefore pooled. Variation decreased on average with age considering all age classes (a linear regression coefficient b = -0.25, p = 6.5x10^-5^, data not shown). Comparison of the three age classes showed that the variation is generally highest for 3–5 years (Table 4).

**Fig 4:**
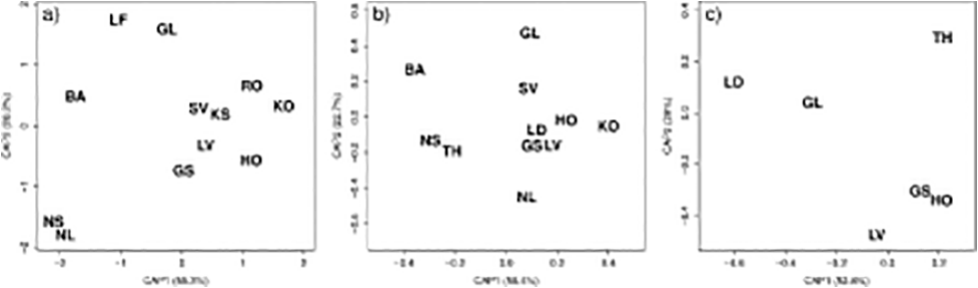
Canonical scores on discriminating axes 1 (CAP1) and 2 (CAP2) for each herring population. BA: Balsfjord, GL: Gloppen, GS: Grimstad, HO: Hovåg, KO: Kragerø, KS: Kilsund, LD: Lindåspollene, LF: Lusterfjord, LV: Lake Landvik, NL: Lofoten, NS: Møre, RO: Risør, SV: Sykkulven and TH: Trondheim in Norway for three age groups: a) 3–5, b) 6–8 and c) 9–12 years (see Table 1 for further details). Black letters represent the mean canonical value for each population, and scores on x- and y-axis show the canonical values which are based on the differences among population.

No interactions were observed for age and populations within age classes 3–5 years, 6–8 years and 9–12 years (p>0.05), however age was significant (p<0.05). Significant differences in otolith shape were detected among all herring populations at ages 3–5, 6–8 and 9–12 years (p<0.001, Table 3), although the differences among populations decreased with age as seen with lower *F*-values (Table 3) and lower CAP values for the older ages (Fig 4B–4C). Examining the position of the populations based on shape variation along the first Canonical axis (Fig 4A–4C), for ages 3–5 years, a pattern emerged with three clusters: the two oceanic populations, Norwegian spring- and autumn spawners, group together (Fig 4A), Sykkulven groups with the populations in southern Norway (Grimstad, Høvåg, Kragerø, Kilsund, Lake Landvik and Risør) while the two populations which occupy the deepest fjords in the study (Lusterfjord and Gloppen) group together. Balsfjord, from the most northerly location, is separate from the rest of the populations. For ages 6–8 years, a similar pattern was observed where the populations from southern Norway (Grimstad, Høvåg, Kragerø, Lake Landvik) group together along with Lindåspollene from western Norway. The Norwegian spring-spawners and Trondheim which occupy similar latitudes in western Norway group together, while populations from Sykkulven, Gloppen and the Norwegian autumn-spawners seem diverged from the rest. Balsfjord again is quite distinct from the rest as was seen for ages 3–5 years. For ages 9–12 years, populations Grimstad, Høvåg and Lake Landvik group together along the first axis, while populations Gloppen, Lindåspollene and Trondheim show no sign of grouping and are quite distinct from the other populations. These results are in accordance with the a priori comparisons (Table 3) where significant differences where found for 3–5 years and 6–8 years in a comparison of Balsfjord vs fjord populations (p<0.001), between populations occupying western and southern Norway for all age groups and also within western Norway (p<0.001). Comparing populations within southern Norway at ages 3–5 and 6–8 years, significant differences in shape where found (p<0.008), while at ages 9–12 populations did not differ (p>0.05). The two oceanic populations, the Norwegian spring- and autumn-spawners, differed each from the fjord populations, both at ages 3–5 and 6–8 (p<0.003).

### Otolith shape and geographical distance

**Fig 5:**
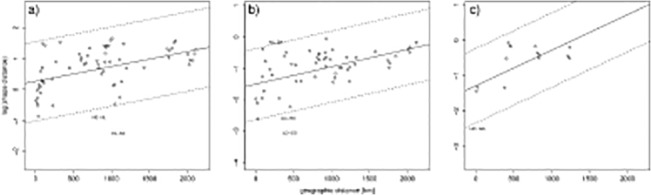
The association of otolith shape with respect to geographic distances in km between sampling areas from Kragerø in southern Norway to Balsfjord in northern Norway. The age groups are: a) 3–5, b) 6–8 and c) 9–12 years. The correlation of the shape distances with geographical distances was for the three age classes: r_3-5y_ = 0.44, r_6-8y_ = 0.66, r_9-12y_ = 0.57, with p<0.001 in all cases, based on 10.000 permutations. A trend line based on linear regression is shown, dotted lines represents two standard deviations of the residuals from the regression line. Population pairs which distances fall outside of the two standard deviations are presented (see Area ID codes in Table 1).

There was a latitudinal gradient along the coastline in otolith shape of the studied populations. Populations found in habitats geographically close to each other were more similar in otolith shape than populations further apart (Fig 5A–5C, r_3-5y_ = 0.44, r_6-8y_ = 0.66, r_9-12y_ = 0.57, p<0.001 for all comparisons based on 10.000 permutations). Few population pairs differed from the overall trend expected by the geographical distance. The oceanic populations were more similar to each other at ages 3–5 years than at the other ages (Fig 5A). One population from western Norway (Sykkulven), showed similarities with one population from southern Norway (Kilsund) and both of these populations had large variance within populations (Table 4). For the age group 6–8 years, Lindåspollene from western Norway showed similarities with Høvåg and Grimstad from southern Norway (Fig 5B), but Lindåspollene had considerably low sample size at these ages. At the same ages, the neighbouring populations, the Norwegian autumn-spawners and Balsfjord in northern Norway deviated more from each other, when considering the geographic distance, than all pairs from ages 6–8 years.

## Discussion

Otolith shape analysis of Atlantic herring in Norwegian waters showed significant variation among the locations studied. In addition, isolation by distance emerged with a latitudinal gradient along the coastline. These morphological differences indicate low dispersal of adult individuals, assuming that the shape is determined at young age, and support even a reproductive isolation among the local herring populations [52]. Our results suggest that the semi-enclosed systems, where the local populations live and breed, are efficient barriers for adult dispersal, which has resulted in diversification of the local fjord populations. Dispersal of larvae cannot though be ruled out if the shape is mainly determined by the environment during early life. A gradient in shape can arise due to the effect of an environmental gradient on the otolith shape.

The significant differences in otolith shape points to limited exchange between the local populations and their oceanic counterparts, but to what degree the oceanic populations interbreed with the local populations is not fully known. The oceanic Norwegian spring-spawners have been found to spawn in the same area as Lindåspollene herring for 50 years and to alter the life-history of the resident population [7], but their otolith shape differs. This observed variation between the oceanic and local populations might be due to the environmental differences encountered by the populations during early life and thus after genetic admixture or larval dispersal. While the local populations are refined in semi-enclosed ecosystems and exhibit relatively stable local conditions, the juveniles of the oceanic populations, which are recruited along the central Norwegian continental shelf, show growth similar to northern populations as they exhibit less growth with decreasing temperature and increasing latitude as they are carried northwards with the coastal current into the Barents Sea [36–39]. Variation in growth rates can cause otolith increments to be deposited differently, where faster growth enhances daily ring deposition and slower growth results in fewer rings, which affects the otolith structure [67–70]. It is therefore likely that differing growth rates are contributing to the shape differences observed among the local populations and the oceanic populations.

Local populations occupying southern Norway and western Norway were more similar in otolith shape to their neighbouring populations than to the more distant populations. This was observed for all the three age intervals tested, even though the number of samples from the oldest age class was limited. Balsfjord herring, from the most northerly location (69°N), was most different in otolith shape compared to the other local populations. Balsfjord herring is likely to be an outlier in our analysis, not only with regard to their geographic position, but also given their genetic similarity with Pacific herring, based on mtDNA [32,53,71]. Balsfjord herring has also been shown to be more similar to Pacific herring in vertebrae number [72] and spawning behaviour [17] than to both local and oceanic Atlantic herring [17,53]. The oceanic populations, the Norwegian spring- and autumn-spawners, were considerably different in otolith shape compared to the other populations, which might be attributed to their higher dispersal capacity compared to the local populations. At the younger ages (3–5 years, Fig 4A), the oceanic populations group together but they become different at older ages (6–8, Fig 4B) as previously reported [8].

Deviations from the overall trend include the variability in the results between the 3–5 year olds and the 6–8 years olds (Fig 4A and 4B). There was similarity in otolith shape of populations from Sykkulven from western Norway and Kilsund from southern Norway (Fig 4A). Lindås from western Norway grouped with Høvåg and Grimstad in southern Norway for ages 6–8 years (Fig 4B). To which extent the overall trend and these deviations can be explained by the particular characteristics of the different populations, is unclear. It might be linked to the temperature differences found along the latitudinal gradient along the Norwegian coast [38], or it might be linked to actual different life history strategies as seen in the growth (length-at-age and asymptotic length), maturity ogives and reproductive effort of these local populations (Table 1) [7,21,23,24,26,27,29,33,34].

In general, fish populations are known to be differently constrained by survival and reproduction trade-offs [73], and differ in size at maturity directly influencing the populations growth rates [74]. Also, otolith shape might be influenced by differing food rations [75]. Hence, the observed deviations and variance at particular age groups may result from single or combined effects of food limitations or temperature differences, even though they may reach their maximum length asymptotically at different ages.

Modifications of the mean otolith shape were detected and differed among populations at three main positions, the excisura major, rostrum and the excisura minor (Fig 2). An interesting pattern emerged where the indentation at the excisura major was most pronounced in the otoliths of the Norwegian spring-spawners which is in line with former studies both from the Northeast Atlantic [8] and the Landvik region in southern Norway [26]. Next to the Norwegian spring-spawners was the other oceanic population in the study, the Norwegian autumn-spawners from Lofoten, then Trondheim herring and Balsfjord herring. Both at the rostrum and the excisura minor area the same pattern was seen, where Balsfjord herring had the most indented shape, next Lusterfjord and then Gloppen. These populations have in common a considerably shorter body length due to slower growth rates for herring which grow up within the fjord ecosystem [21,30,31] (Table 1), which could be contributing to these differences. Herring populations west of the British Isles which also mature at a younger age, show considerable size differences and differing growth rates in comparison to the populations in the northern part of the NE-Atlantic [76] and variation in otolith shape [8]. As mentioned, the growth rate differences among these populations might be contributing to the shape differences observed [67–70].

The multivariate analysis showed temporal stability in otolith shape among the populations with more than one sampling year from Balsfjord, Gloppen, Risør and Sykkulven. To our knowledge, this study is the first to report temporal stability in otolith shape among herring populations, further proving the usefulness of otolith shape as a marker for population discrimination of herring [8].

For pelagic species with high gene flow, the present results emphasize the importance of not only focusing on genetic variability but also to take into account the identification of phenotypic stocks to ensure sustainable fisheries and conservation of the species. Several of the smaller local populations observed have unique life history characteristics [7,21,23,24,26,27,29,33,34] and therefore differ in their response to exploitation. From the management point of view and by definition, north of 62°, herring are managed as the Norwegian spring-spawners with the exception of Trondheimsfjord herring which are protected. South of 62°, only one local population, Lindåspollene herring, is protected, while other herring are caught and managed as North Sea and western Baltic Sea herring. The differences in otolith shape found in the present study demonstrate that several of the local populations south of 62° are diverse. It is apparent that the herring population structure in Norway is complex which indicates that new management strategies, taking into account the diversification of these smaller populations, to protect the biodiversity, might be warranted.
