# Correction: Latitudinal Gradient in Otolith Shape among Local Populations of Atlantic Herring (*Clupea harengus* L.) in Norway

**DOI:** 10.1371/journal.pone.0145900

**Published:** 2015-12-23

**Authors:** The PLOS ONE Staff

Figs 4 and 5 are of low resolution; the publisher apologizes for this error. Please see the corrected Figs 4 and 5 here.

**Fig 4:**
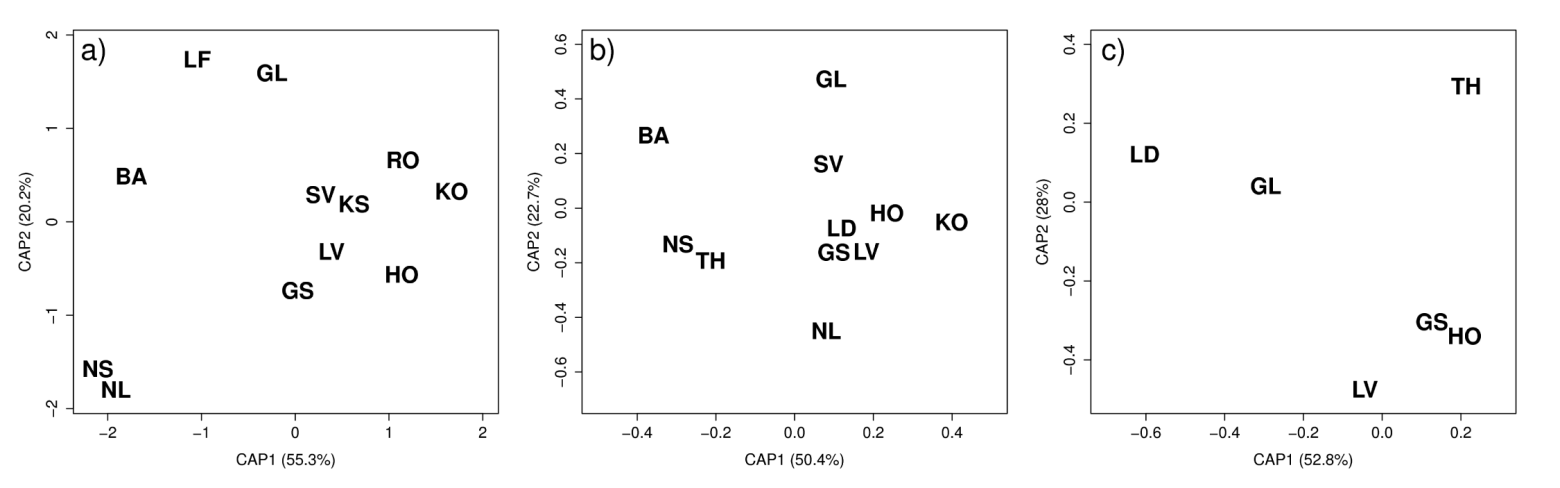
Canonical scores on discriminating axes 1 (CAP1) and 2 (CAP2) for each herring population. BA: Balsfjord, GL: Gloppen, GS: Grimstad, HO: Hovåg, KO: Kragerø, KS: Kilsund, LD: Lindåspollene, LF: Lusterfjord, LV: Lake Landvik, NL: Lofoten, NS: Møre, RO: Risør, SV: Sykkulven and TH: Trondheim in Norway for three age groups: a) 3–5, b) 6–8 and c) 9–12 years (see Table 1 for further details). Black letters represent the mean canonical value for each population, and scores on x- and y-axis show the canonical values which are based on the differences among population.

**Fig 5:**
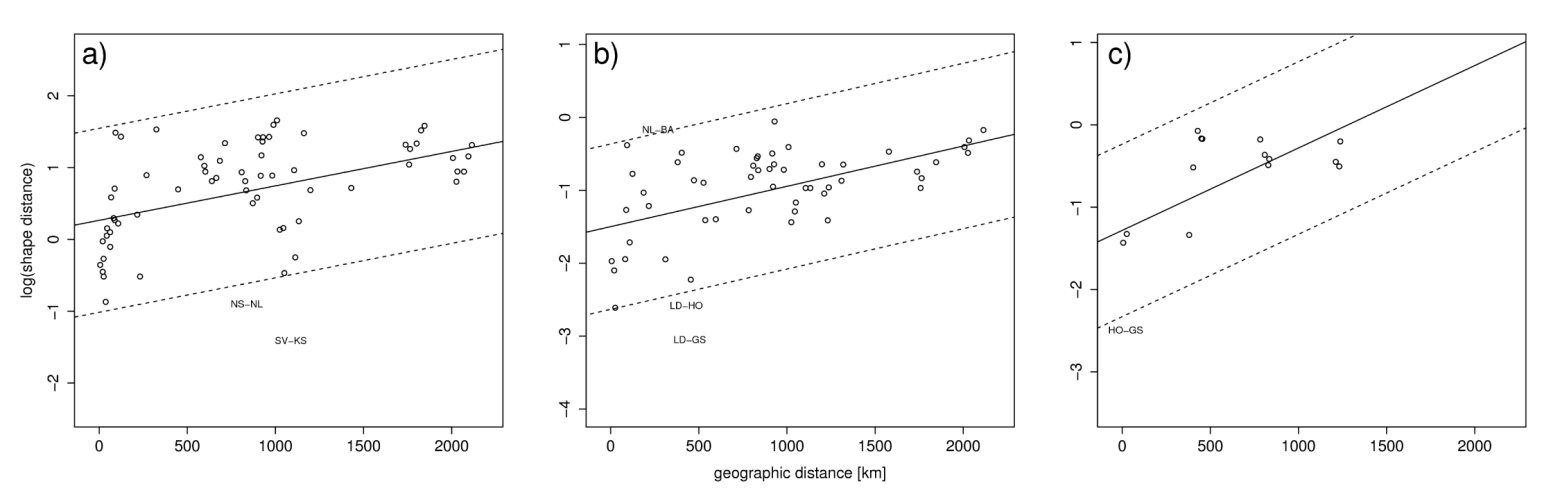
The association of otolith shape with respect to geographic distances in km between sampling areas from Kragerø in southern Norway to Balsfjord in northern Norway. The age groups are: a) 3–5, b) 6–8 and c) 9–12 years. The correlation of the shape distances with geographical distances was for the three age classes: r_3-5y_ = 0.44, r_6-8y_ = 0.66, r_9-12y_ = 0.57, with p<0.001 in all cases, based on 10.000 permutations. A trend line based on linear regression is shown, dotted lines represents two standard deviations of the residuals from the regression line. Population pairs which distances fall outside of the two standard deviations are presented (see Area ID codes in Table 1).

## References

[1] Libungan LA, Slotte A, Husebø Å, Godiksen JA, Pálsson S (2015) Latitudinal Gradient in Otolith Shape among Local Populations of Atlantic Herring (*Clupea harengus* L.) in Norway. PLoS ONE 10(6): e0130847 doi:10.1371/journal.pone.0130847 26101885 10.1371/journal.pone.0130847 PMC4478005

